# Census tract socioeconomic indicators and COVID-19-associated hospitalization rates—COVID-NET surveillance areas in 14 states, March 1–April 30, 2020

**DOI:** 10.1371/journal.pone.0257622

**Published:** 2021-09-24

**Authors:** Jonathan M. Wortham, Seth A. Meador, James L. Hadler, Kimberly Yousey-Hindes, Isaac See, Michael Whitaker, Alissa O’Halloran, Jennifer Milucky, Shua J. Chai, Arthur Reingold, Nisha B. Alden, Breanna Kawasaki, Evan J. Anderson, Kyle P. Openo, Andrew Weigel, Maya L. Monroe, Patricia A. Ryan, Sue Kim, Libby Reeg, Ruth Lynfield, Melissa McMahon, Daniel M. Sosin, Nancy Eisenberg, Adam Rowe, Grant Barney, Nancy M. Bennett, Sophrena Bushey, Laurie M. Billing, Jess Shiltz, Melissa Sutton, Nicole West, H. Keipp Talbot, William Schaffner, Keegan McCaffrey, Melanie Spencer, Anita K. Kambhampati, Onika Anglin, Alexandra M. Piasecki, Rachel Holstein, Aron J. Hall, Alicia M. Fry, Shikha Garg, Lindsay Kim

**Affiliations:** 1 CDC COVID-NET Team, Atlanta, GA, United States of America; 2 US Public Health Service, United States of America; 3 Connecticut Emerging Infections Program, Yale School of Public Health, New Haven, CT, United States of America; 4 California Emerging Infections Program, Oakland, CA, United States of America; 5 CDC Career Epidemiology Field Officer, Oakland, CA, United States of America; 6 Colorado Department of Public Health and Environment, Denver, CO, United States of America; 7 Emerging Infections Program, Georgia Department of Public Health, Atlanta, GA, United States of America; 8 Veterans Affairs Medical Center, Atlanta, GA, United States of America; 9 Division of Infectious Diseases, School of Medicine, Emory University, Atlanta, GA, United States of America; 10 Iowa Department of Public Health, Des Moines, IA, United States of America; 11 Maryland Department of Health, Baltimore, MD, United States of America; 12 Michigan Department of Health and Human Services, Lansing, MI, United States of America; 13 Minnesota Department of Health, St. Paul, MN, United States of America; 14 New Mexico Department of Health, Santa Fe, NM, United States of America; 15 University of New Mexico Emerging Infections Program, Albuquerque, NM, United States of America; 16 New York State Department of Health, Albany, NY, United States of America; 17 University of Rochester School of Medicine and Dentistry, Rochester, NY, United States of America; 18 Ohio Department of Health, Columbus, OH, United States of America; 19 Public Health Division, Oregon Health Authority, Portland, OR, United States of America; 20 Vanderbilt University Medical Center, Nashville, TN, United States of America; 21 Utah Department of Health, Salt Lake City, UT, United States of America; 22 Salt Lake County Health Department, Salt Lake City, UT, United States of America; University of Utah, UNITED STATES

## Abstract

**Objectives:**

Some studies suggested more COVID-19-associated hospitalizations among racial and ethnic minorities. To inform public health practice, the COVID-19-associated Hospitalization Surveillance Network (COVID-NET) quantified associations between race/ethnicity, census tract socioeconomic indicators, and COVID-19-associated hospitalization rates.

**Methods:**

Using data from COVID-NET population-based surveillance reported during March 1–April 30, 2020 along with socioeconomic and denominator data from the US Census Bureau, we calculated COVID-19-associated hospitalization rates by racial/ethnic and census tract-level socioeconomic strata.

**Results:**

Among 16,000 COVID-19-associated hospitalizations, 34.8% occurred among non-Hispanic White (White) persons, 36.3% among non-Hispanic Black (Black) persons, and 18.2% among Hispanic or Latino (Hispanic) persons. Age-adjusted COVID-19-associated hospitalization rate were 151.6 (95% Confidence Interval (CI): 147.1–156.1) in census tracts with >15.2%–83.2% of persons living below the federal poverty level (high-poverty census tracts) and 75.5 (95% CI: 72.9–78.1) in census tracts with 0%–4.9% of persons living below the federal poverty level (low-poverty census tracts). Among White, Black, and Hispanic persons living in high-poverty census tracts, age-adjusted hospitalization rates were 120.3 (95% CI: 112.3–128.2), 252.2 (95% CI: 241.4–263.0), and 341.1 (95% CI: 317.3–365.0), respectively, compared with 58.2 (95% CI: 55.4–61.1), 304.0 (95%: 282.4–325.6), and 540.3 (95% CI: 477.0–603.6), respectively, in low-poverty census tracts.

**Conclusions:**

Overall, COVID-19-associated hospitalization rates were highest in high-poverty census tracts, but rates among Black and Hispanic persons were high regardless of poverty level. Public health practitioners must ensure mitigation measures and vaccination campaigns address needs of racial/ethnic minority groups and people living in high-poverty census tracts.

## Background

Since SARS-CoV-2, the novel coronavirus that causes coronavirus disease 2019 (COVID-19), was first detected in December 2019 [1], more than 100 million cases have been reported worldwide, including more than 25 million in the United States [2, 3]. While most persons infected with SARS-CoV-2 are asymptomatic or develop only mild symptoms, approximately 14% require hospitalization [4]. Older age, especially age ≥65 years, and underlying medical conditions are risk factors for COVID-19-associated hospitalizations [5, 6] and mortality [7–9]. In addition, some studies have documented increased morbidity in certain racial/ethnic groups [6, 10, 11]. Some have proposed that these observations are the result of underlying socioeconomic characteristics [12], which have been implicated in morbidity associated with many infectious conditions [13–17].

Understanding relationships between socioeconomic characteristics and COVID-19-associated hospitalizations could inform the ongoing public health response to COVID-19 in at least two ways. First, quantifying relationships between COVID-19-associated hospitalizations and socioeconomic indicators could demonstrate the utility and facilitate the use of these known characteristics in local and national public health practice. Second, further characterization of the relationship between socioeconomic indicators and hospitalization rates might provide better insight into the degree to which racial/ethnic disparities in COVID-19-associated hospitalizations result from underlying differences in socioeconomic status that often overlap with race and ethnicity in the United States [18]. This knowledge might guide efforts targeted towards underlying causes of racial/ethnic disparities in COVID-19-associated hospitalization rates. Therefore, we sought to examine associations between COVID-19-associated hospitalization rates, census tract-level socioeconomic status indicators, and race/ethnicity.

## Methods

To obtain counts of COVID-19-associated hospitalizations, we used data from the COVID-19-Associated Hospitalization Surveillance Network (COVID-NET), which conducts population-based surveillance in 99 counties in 14 states (California, Colorado, Connecticut, Georgia, Iowa, Maryland, Michigan, Minnesota, New Mexico, New York, Ohio, Oregon, Tennessee, and Utah). Approximately 32 million people, or 10% of the U.S. population, live within the COVID-NET surveillance area. COVID-NET defines a case as a resident of the surveillance catchment area with a positive test for SARS-CoV-2 within 14 days of hospitalization. Trained public health surveillance staff proactively query healthcare facilities and public health reporting systems to ensure complete case ascertainment; using a standardized case report form, these staff collect detailed case demographic and clinical data through medical chart review. To verify that cases reside within the surveillance area, COVID-NET obtains residential addresses. Using available software, surveillance personnel obtained geographic coordinates of residential addresses and matched them with census tracts (i.e., geocode) [19]. In this analysis, we included cases among persons ≥18 years, including those residing in long-term care facilities and correctional facilities. We excluded cases with missing addresses, addresses that could not be geocoded, and those that represented post office boxes. We also excluded cases among persons experiencing homelessness.

To examine socioeconomic indicators, we used data from the 2018 American Community Survey (ACS), which is conducted by the U.S. Census Bureau and captures census tract-level socioeconomic indicators, such as poverty, employment information, economic information, and educational attainment [20, 21]. The year 2018 was the most recent survey year for which the tract-level data necessary for our analysis were available. The ACS is sent to a sample of addresses (about 3.5 million) in the 50 states, District of Columbia, and Puerto Rico. We considered all census tracts within the surveillance catchment area and divided these census tracts into quartiles based on each of four indicators: the percentage of persons living below the federal poverty level, the percentage of employed persons aged ≥16 years working in service industry occupations, the percentage of persons commuting to work using public transportation, and the percentage of persons aged ≥25 years without bachelors’ degrees. Denominators were total populations residing in each socioeconomic indicator quartile and appropriate racial/ethnic/age group, using data from the 2010 decennial U.S. Census [22]. Numerators were the total numbers of COVID-NET cases residing in census tracts in the socioeconomic indicator quartiles; rate calculations were performed using these numerators and the corresponding indicator-specific denominator [22]. We used Poisson regression to calculate 95% confidence intervals for hospitalization rates. Dividing indicators into quartiles facilitated assessment of relationships between socioeconomic several indicator levels and COVID-19-associated hospitalization rates. Additionally, dividing indicators into quartiles facilitated comparisons across socioeconomic indicators, especially with ones that are not as commonly considered (e.g., public transportation use) in health studies. To assess effect modification by race/ethnicity (i.e., differences in the association between census tract poverty and COVID-19-associated hospitalization rates), we stratified the analysis by 3 racial/ethnic groups, non-Hispanic Black (Black), non-Hispanic White (White), and Hispanic/Latino (Hispanic); persons not reported as Hispanic were considered non-Hispanic. Because age is a risk factor for hospitalization and severe COVID-19, we also stratified analyses by age (i.e., compared rates among persons aged 18–64 with those among persons aged ≥65 years) and calculated age-adjusted rates to account for differences in the age distribution among different racial/ethnic and socioeconomic groups in the COVID-NET catchment area using population denominators from the 2010 decennial U.S. Census and age strata 18–49, 50–64, and ≥65 years[21]. All analyses were performed using SAS 9.4.

This activity was reviewed by the United States Centers for Disease Control and Prevention (CDC) and was conducted consistent with applicable federal law and CDC policy (See e.g., 45 C.F.R. part 46, 21 C.F.R. part 56; 42 U.S.C. §241(d); 5 U.S.C. §552a; 44 U.S.C. §3501 et seq). Participating sites obtained approval for COVID-NET surveillance from their respective state and local IRBs as required.

## Results

During March 1–April 30, 2020, COVID-NET captured 18,540 cases among patients aged ≥18 years. Of these, 2,540 (14%) cases were excluded from the analysis because there was no residential address (n = 812), the address could not be assigned to a census tract (n = 1,351), represented a post office box (n = 285), or occurred in a patient experiencing homelessness (n = 92). Therefore, geocoded residential addresses were available for 16,000 (86%) cases; of these, 1,863 (11%) resided in long-term care facilities and 14 (<1%) resided in correctional facilities. Homeless status was undetermined for 7,455 (47%) cases.

Of the 16,000 cases with geocoded residential addresses, 7,488 (46.8%) occurred in persons ≥65 years old (Table 1). Black persons comprised 36.3%, White persons 34.8%, Hispanic persons 18.2%, non-Hispanic, Asian or Pacific Islander (Asian) persons 4.6%, and non-Hispanic American Indian/Alaska Native persons 0.6%. Race/ethnicity was unknown or unavailable pending further record review for 1,442 (9.0%) cases.

**Table 1:** Characteristics of adult patients (≥18 years old) hospitalized with COVID-19 and available geocoding information—COVID-NET catchment areas in 14 states, March 1–April 30, 2020.

Characteristic	n (%)
	N = 16,000
**Age group (years)**	
18–29 years	638 (4.0)
30–39 years	1,223 (7.6)
40–49 years	1,899 (11.9)
50–64 years	4,752 (29.7)
≥65 years	7,488 (46.8)
**Sex**	
Male	8,497 (53.1)
Female	7,502 (46.9)
**Race/ethnicity**	
Hispanic or Latino	2,800 (18.2)
White, NH	5,353 (34.8)
Black, NH	5,572 (36.3)
American Indian/Alaska Native, NH	98 (0.6)
Asian or Pacific Islander, NH	700 (4.6)
Multiracial, NH	35 (0.2)
Unknown or Missing	1,442 (9.0)
**Percentage of persons living below federal poverty level in census tracts of residence (Quartile, %)**	
Highest quartile, high-poverty tracts (>15.2%–83.2%)	4,473 (28.0)
Second quartile (>8.6%–15.2%)	4,279 (26.7)
Third quartile (>4.9%–8.6%)	3,950 (24.7)
Lowest quartile, low-poverty tracts (0%–4.9%)	3,285 (20.5)
Unknown	13 (<0.1)
**Percentage of service-industry workers in census tracts of residence (Quartile, %)**	
Highest quartile (>22.3%–58.3%)	5,296 (33.1)
Second quartile (>16.6%–22.3%)	3,916 (24.5)
Third quartile (>12.2%–16.6%)	3,651 (22.8)
Lowest quartile (0%–12.2%)	3,124 (19.5)
Unknown	13 (<0.1)
**Percentage of persons commuting using public transportation in census tracts of residence (Quartile, %)**	
Highest quartile (>9.7%–70.6%)	4,932 (30.8)
Second quartile (>3.9%–9.7%)	4,222 (26.4)
Third quartile (>1.1%–3.9%)	3,637 (22.7)
Lowest quartile (0%–1.1%)	3,196 (20.0)
Unknown	13 (<0.1)
**Proportion of persons aged ≥25 years without bachelor’s degrees in census tracts of residence (Quartile, %)**	
Highest quartile (>86.2%–99.2%)	4,801 (30.0)
Second quartile (>78.6%–86.2%)	4,421 (27.6)
Third quartile (>70.2%–78.6%)	3,913 (24.5)
Lowest quartile (44.4%–70.2%)	2,863 (17.9)
Unknown	2 (<0.1)

Abbreviations: NH = non-Hispanic.

Of the 16,000 COVID-19-associated hospitalizations with geocoded addresses, 4,473 (28.0%) occurred among persons living in the quartile of census tracts with the highest percentages of persons living below the federal poverty level (high-poverty census tracts), 5,296 (33.1%) occurred among persons living in the quartile of census tracts with the highest percentages of service industry workers, 4,932 (30.8%) cases occurred among persons living in the quartile of census tracts with the highest percentages of commuters using public transportation, and 4,801 (30.0%) cases occurred among persons living in the quartile census tracts with the highest percentages of persons aged ≥25 years without bachelor’s degrees (Table 1).

**Fig 1:**
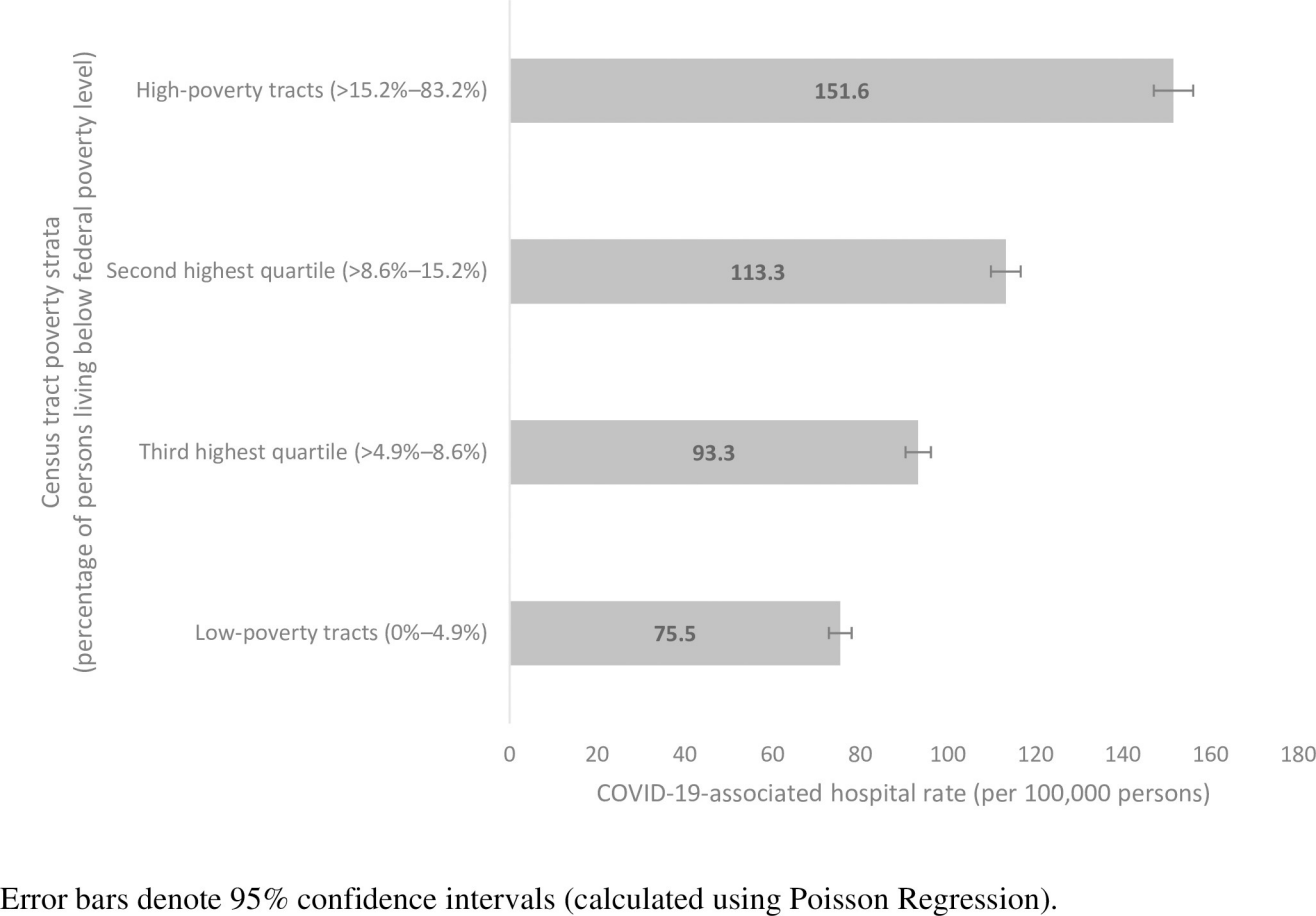
Age-adjusted COVID-19-associated hospitalization rates by census tract-level poverty strata (quartile)—COVID-NET catchment areas in 14 states, March–April 2020.

Overall, unadjusted COVID-19-associated hospitalization rates were highest in high-poverty census tracts. Unadjusted COVID-19-associated hospitalization rates were 133.5 per 100,000 persons (95% confidence interval (CI): 129.6–137.4) in high-poverty census tracts and 80.1 (95% CI: 77.4–82.9) in low-poverty census tracts (i.e., those with 0%–4.9%, the lowest quartile, of persons living below the federal poverty level) (Table 2). Age-adjusted COVID-19-associated hospitalization rates by poverty level followed similar trends (Fig 1).

**Table 2:** Unadjusted COVID-19-associated hospitalization rates, by socioeconomic strata within census tracts—COVID-NET catchment areas in 14 states, March–April 2020.

Indicator^1^	Quartile	Unadjusted COVID-19-associated hospitalization rate^2^^,^ ^3^, (95% Confidence Intervals)	Rate Ratio
Percentage of persons living below federal poverty level in census tracts of residence	Highest: >15.2%–83.2%	133.5 (129.6–137.4)	1.7 (1.6–1.8)
	Second: >8.6%–15.2%	110.1 (106.8–113.4)	1.4 (1.3–1.5)
	Third: >4.9%–8.6%	97.6 (94.6–100.7)	1.2 (1.1–1.3)
	Lowest: 0%–4.9%	80.1 (77.4–82.9)	Referent
Percentage of service-industry workers in census tracts of residence	Highest: >22.3%–58.3%	155.5 (151.4–159.7)	2.0 (1.9–2.1)
	Second: >16.6%–22.3%	100.3 (97.2–103.5)	1.3 (1.2–1.4)
	Third: >12.2%–16.6%	88.9 (86.1–91.9)	1.1 (1.0–1.2)
	Lowest: 0%–12.2%	78.7 (75.9–81.5)	Referent
Percentage of persons commuting using public transportation in census tracts of residence	Highest: >9.7%–70.6%	142.9 (139.0–146.9)	1.8 (1.7–1.9)
	Second: >3.9%–9.7%	112.3 (109.0–115.8)	1.5 (1.4–1.6)
	Third: >1.1%–3.9%	89.8 (86.9–92.8)	1.2 (1.1–1.3)
	Lowest: 0%–1.1%	77.4 (74.8–80.1)	Referent
Proportion of persons aged ≥25 years without bachelor’s degrees in census tracts of residence	Highest: 86.2%–99.2%	145.5 (141.4–149.6)	2.0 (1.9–2.1)
	Second: >78.6%–86.2%	111.5 (108.3–114.9)	1.6 (1.5–1.7)
	Third: >70.2%–78.6%	95.1 (92.2–98.1)	1.3 (1.2–1.4)
	Lowest: 44.4%–70.2%	71.2 (68.6–73.8)	Referent

^1^ Source: 2018 American Community Survey, US Census Bureau.

^2^ Defined as the number of COVID-19-associated hospitalizations captured within census tracts within the surveillance catchment area having socioeconomic indicators within the given strata area divided by the total number of persons aged ≥18 years living within the census tracts in the surveillance catchment area having socioeconomic indicators within the given strata according to the 2010 decennial census.

^3^ Rate per 100,000 persons.

**Fig 2:**
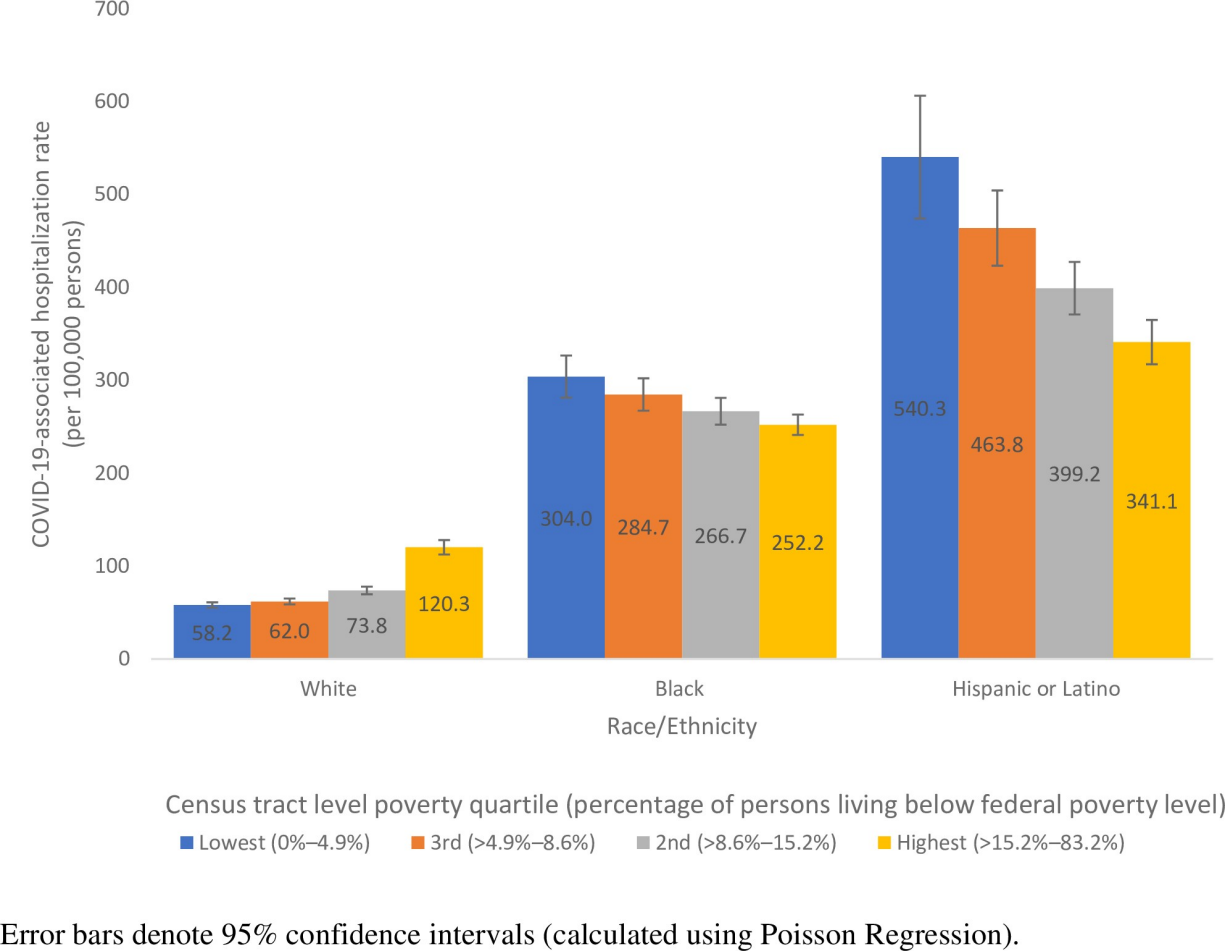
Age adjusted COVID-19-associated hospitalization rates by census tract-level poverty strata (quartiles) and race/ethnicity—COVID-NET catchment areas in 14 states, March–April 2020.

While overall age-adjusted hospitalization rates were associated with high-poverty census tracts, the relationships between COVID-19-associated hospitalizations and census tract-level poverty differed by race/ethnicity. Among White persons, age-adjusted COVID-19-associated hospitalization rates were lowest in low-poverty census tracts and increased with increasing census tract poverty level (Fig 2). While the absolute rates in all racial/ethnic groups were highest among those aged ≥65 years regardless of socioeconomic status, COVID-19-associated hospitalization rates increased with census tract poverty level among White persons, whether aged 18–64 or ≥65 years (S1 Fig).

Among Black and Hispanic persons, trends were different. Age-adjusted COVID-19-associated hospitalization rates per 100,000 persons were 304.0 (95% CI: 282.4–325.6) and 540.3 (95% CI: 477.0–603.6), respectively, for Black and Hispanic persons residing in low-poverty census tracts and 252.2 (95% CI: 241.4–263.0) and 341.1 (95% CI: 317.3–365.0), respectively, for Black and Hispanic persons residing in high-poverty census tracts. Age-adjusted rates in both racial/ethnic groups decreased with increasing census tract poverty level (Fig 2). Nonetheless, there were no statistically significant differences in hospitalization rates between census tracts representing the 3 lowest poverty quartiles for either racial/ethnic group (Fig 2).

**Fig 3:**
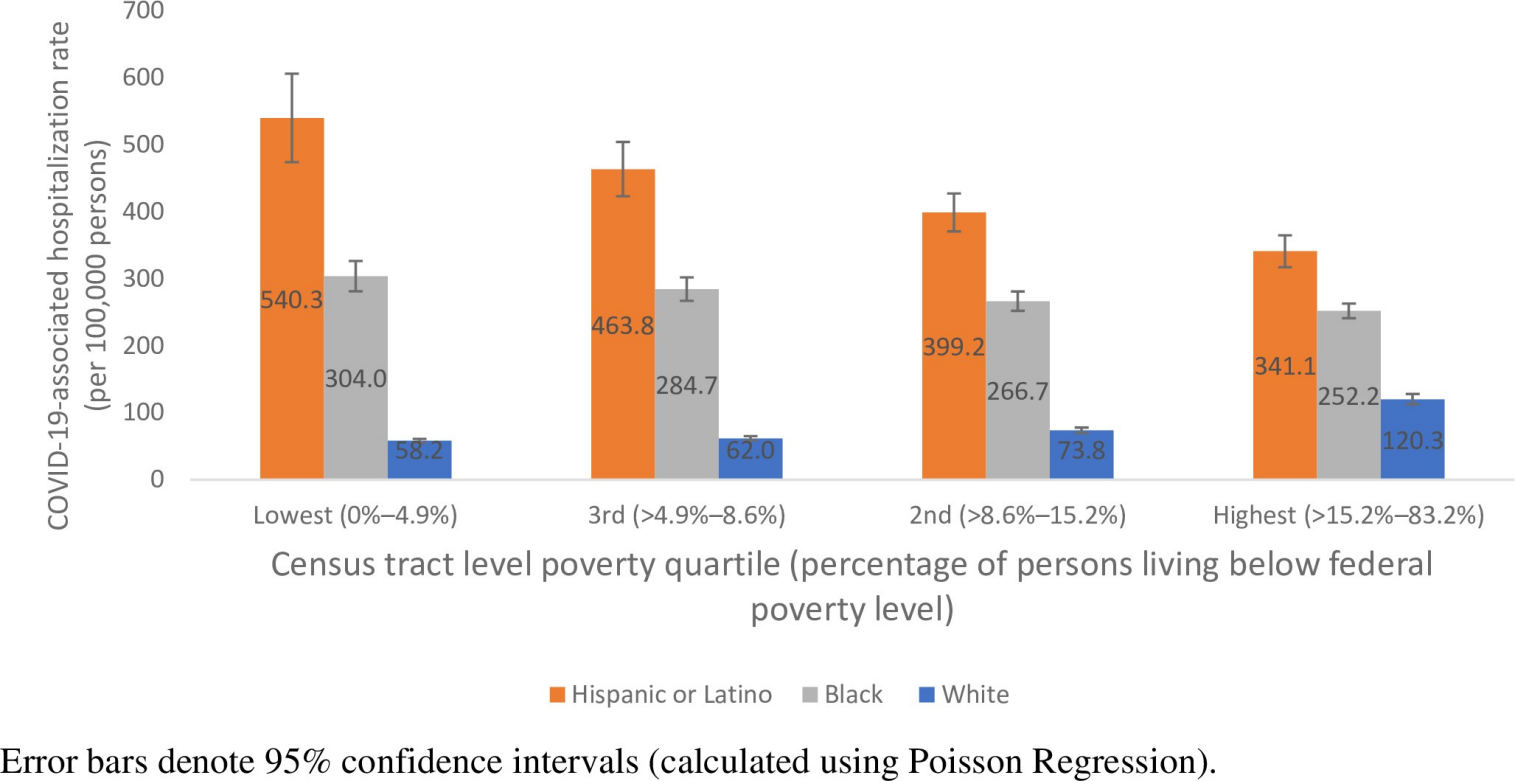
Age-adjusted COVID-19-associated hospitalization rates by census tract-level poverty strata (quartiles) and race/ethnicity—COVID-NET catchment areas in 14 states, March–April 2020.

The magnitude of the differences in COVID-19-associated hospitalization rates between racial/ethnic groups varied by census tract poverty quartile (Fig 3). Differences between age-adjusted, race/ethnicity-specific COVID-19-associated hospitalization rates were largest in low-poverty census tracts; rates among Hispanic persons and Black persons were 9.2 and 5.3 times, respectively, greater than rates among White persons in low-poverty census tracts. Differences in age-adjusted, race/ethnicity-specific COVID-19-associated hospitalization rates decreased as census tract poverty increased. While differences in age-adjusted, race/ethnicity specific rates persisted despite poverty level, these differences were smallest in high-poverty census tracts; rates among Hispanic persons and Black persons were 2.9 and 2.2 times, respectively, greater than rates among White persons in high-poverty census tracts. Age-stratified rates followed similar trends (S1 Table).

COVID-19-associated hospitalization rates were also associated with the percentage of service-industry workers residing within census tracts. Of the 5,296 hospitalized cases living in census tracts with the highest percentages of service industry workers, 2,995 (56.6%) also lived in high-poverty census tracts. In census tracts with the highest quartile of service industry workers, the COVID-19-associated hospitalization rate was 155.5 (95% CI: 151.4–159.7) per 100,000 persons compared with a rate of 78.7 (95% CI: 75.9–81.5) in census tracts with the lowest quartile of service industry workers (Table 2).

Similarly, COVID-19-associated hospitalization rates were associated with the percentage of persons within census tracts who used public transportation. Of the 4,932 hospitalized cases living in census tracts with the highest percentages of people who used public transportation, 2,087 (42.3%) also lived in high poverty census tracts. In census tracts with the highest quartile of commuters using public transportation, the COVID-19-associated hospitalization rate was 142.9 (95% CI: 139.0–146.9) per 100,000 persons compared with 77.4 (95% CI: 74.8–80.1) in census tracts with the lowest quartile of commuters using public transportation (Table 2).

COVID-19-associated hospitalization rates were also associated with the percentages of persons aged ≥25 years without bachelor’s degrees. In census tracts with percentages of persons aged ≥25 years without bachelor’s degrees within the highest quartile, the COVID-19-associated hospitalization rate was 145.5 (95% CI: 141.4–149.6) per 100,000 persons compared with 71.2 (95% CI: 68.6–73.8) in census tracts in the lowest quartile (Table 2).

## Discussion

COVID-19-associated hospitalization rates were associated with four census tract-level socioeconomic indicators. Hospitalization rates were highest in high-poverty census tracts and those with the highest percentages of service industry workers, commuters using public transportation, and persons without bachelor’s degrees. Nonetheless, associations between hospitalization rates and census tract poverty differed between racial/ethnic groups; among White persons, lower census tract poverty was associated with higher hospitalization rates. Because hospitalization rates among Black and Hispanic persons were high regardless of census tract poverty, racial/ethnic disparities in hospitalization rates were largest in low-poverty census tracts.

Race/ethnicity and area-based socioeconomic indicators might identify groups of persons who are at increased risk of becoming infected with SARS-CoV-2. The trends in COVID-19-associated hospitalization rates might result from racial/ethnic and socioeconomic differences in SARS-CoV-2 infection secondary to differences in the ability to adhere to mitigation recommendations, such as stay-at-home orders, while fulfilling essential responsibilities, such as employment [23]. During March–April 2020, mitigation measures decreased risk of becoming infected with SARS-CoV-2 by reducing the number of persons who share airspace. During this time, 42 states instituted stay-at-home orders; implementation of these orders varied by location. Among states with COVID-NET surveillance catchment areas, California implemented the first stay-at-home orders on March 19 [24]. Twelve of the 13 other states implemented stay-at-home orders during March 19–April 2; Iowa did not implement stay-at-home orders during March–April 2020 [24]. Therefore, the effects of stay-at-home orders on hospitalization rates might have differed across COVID-NET sites. Nonetheless, because all of these orders allowed essential work to continue, disproportionately high COVID-19-associated hospitalization rates in census tracts with lower socioeconomic indicators and among racial/ethnic minorities might represent cases in essential workers, particularly if these persons did not have access to properly fitted personal protective equipment [25, 26]. Indeed, COVID-19-associated hospitalization rates were associated with the proportion of workers in service industry occupations; many service industry occupations, such as restaurant work, require working onsite and sharing airspace with others. Similarly, essential workers being exposed during commutes could explain the association between hospitalization rates and commuters using public transportation. Other data also suggest that the demand for public transportation during pandemic-related lockdowns occurred in communities serving essential workers and racial/ethnic minorities, the same two groups with increased hospitalization rates in this analysis [27]. Another possibility is that these two socioeconomic indicators, the percentage of service industry workers and the percentage of commuters using public transportation, are associated with poverty (or race/ethnicity) and their associated hospitalization risks. It is also possible that these findings are influenced by the demographics of the COVID-NET sites most affected by the pandemic during this period. Other structural factors, such as differences in employment opportunity opportunities or neighborhood-specific high-risk exposures, might also account for the socioeconomic and racial/ethnic disparities in COVID-19-associated hospitalization rates.

Alternatively, the findings from this analysis might represent some racial/ethnic and socioeconomic differences in hospitalization risk after infection. Racial/ethnic differences in prevalence of underlying medical conditions that confer increased risk of hospitalization after infection, such as diabetes, might contribute to the findings of this analysis [9, 28, 29]. Similarly, higher prevalence of underlying medical conditions that confer increased risk of hospitalization with COVID-19 has been associated with lower census tract-level socioeconomic indicators and geographic region of the country [29, 30].

COVID-19-associated hospitalization rates were associated with census tract poverty among White persons, but not other racial/ethnic groups. In Black and Hispanic persons, associations between census tract poverty and COVID-19-associated hospitalization rates were more challenging to explain; rates were highest in census tracts in low-poverty census tracts. At least three factors might have contributed to this observation. First, social networks among Black and Hispanic persons might span socioeconomic strata more often than networks among White persons [31]. If true within COVID-NET catchment areas, SARS-CoV-2 transmission chains involving Black and Hispanic persons might involve persons living in census tracts within different poverty quartiles more often and could explain some of the different relationships between census tract poverty and hospitalization rates among Black and Hispanic persons. A second potential explanation is that prevalence of underlying medical conditions that confer increased risk of hospitalization after SARS-CoV-2 infection is highest among racial/ethnic minorities in the highest socioeconomic strata, resulting in higher hospitalization rates among Black and Hispanic persons in this low-poverty quartile compared with rates among White residents of the same census tracts [32]. Another possibility is that laboratory testing for SARS-CoV-2 infection, which was required to meet the COVID-NET case definition, was performed in a differential manner with respect to race/ethnicity or socioeconomic status due to variations in medical practice or scarcity of test kits [33, 34]. Systematic studies of these non-socioeconomic reasons for these racial/ethnic differences might further inform the interpretation of these data. Finally, racial/ethnic or socioeconomic differences in access to healthcare, care seeking, or admissions decisions could also contribute to these findings [35].

This analysis is subject to limitations. This analysis examined relationships between census tract-level socioeconomic indicators, not personal indicators, and hospitalization rates. Because there is likely heterogeneity within census tracts with regards to income, education, occupation, and public transportation use, some individuals might have been mischaracterized with respect to their personal status with respect to these indicators. Therefore, this analysis cannot measure associations between personal socioeconomic characteristics and hospitalization rates. However, associations between area-level socioeconomic indicators and hospitalization rates might be more useful for local and national health systems planning purposes. For example, the association between area-level indicators could be used to target interventions, such as vaccination campaigns, towards neighborhoods likely to have higher COVID-19-associated hospitalization rates. Second, these data were obtained from a discrete surveillance area during the early part of the pandemic in the United States; therefore, the generalizability of these findings across geography or time are unknown because the number of COVID-19 cases has increased since April 2020, and the epidemiology could be different with respect to case characteristics, geography, or time. Nonetheless, data from this population-based surveillance system from this period, during which a substantial number of states and localities issued stay-at-home orders, are still important, because the findings might be helpful to consider when predicting the impact of future stay-at-home orders for COVID-19 or another disease with similar transmission dynamics. Third, during the analysis period, March–April 2020, testing for SARS-CoV-2, which was required to meet the COVID-NET case definition, was relatively limited and often performed at the discretion of treating medical teams [34]. Therefore, because some persons with COVID-19 might not have been tested, we might have underestimated COVID-19-associated hospitalizations. Additionally, if testing was associated with socioeconomic indicators, we might have mischaracterized associations between these indicators and hospitalization rates. Fourth, we had to exclude persons experiencing homelessness and those with only post office boxes from the analysis; because it is likely that these persons were unevenly distributed with respect to census tract socioeconomic indicators, we might have underestimated COVID-19-associated hospitalization rates in census tracts with disproportionate numbers of persons who fit these descriptions (e.g., rural census tracts where more people have post office boxes and high-poverty census tracts with more people experiencing homelessness). Finally, because we included persons in long-term care facilities and correctional facilities, outbreaks in these congregate settings might have influenced these findings. Nonetheless, one strength of population-based surveillance is the ability to report rates of disease; because these persons are represented in census denominators, including them among cases facilitates the examination of the relationship between census tract socioeconomic indicators and hospitalization rates.

Despite these limitations, collecting geocoded data can provide critically important information for public health practice. Not only might these data be helpful in forecasting demands on health systems during the current COVID-19 pandemic and other infectious disease outbreaks with similar transmission dynamics, but they might also guide efforts to tailor public health interventions, such as campaigns for mask wearing and vaccine distribution, towards groups with high hospitalization rates. Consistent with other work, this analysis highlights a statistically significant association between census tract-level socioeconomic indicators and corresponding COVID-19-associated hospitalization rates [36]. Despite adjustment for socioeconomic status, racial/ethnic disparities in COVID-19-associated hospitalization rates persisted; even in low-poverty census tracts, age-adjusted hospitalization rates among Hispanic and Black persons were 9 and 5 times, respectively, rates among White persons. Public health practitioners must ensure the mitigation measures and vaccination campaigns address the social, behavioral, and medical needs of racial/ethnic minority groups and people living in census tracts with lower socioeconomic indicators.

## Supporting information

S1 Fig COVID-19-associated hospitalization rates by census tract-level poverty strata (quartiles), race/ethnicity, and age groups—COVID-NET catchment areas in 14 states, March–April 2020. (TIF)

S1 Table COVID-19-associated hospitalization rates by census tract-level poverty strata (quartiles), race/ethnicity, and age groups—COVID-NET catchment areas in 14 states, March–April 2020. (TIF)
