# Supplementary figures and images for: Census tract socioeconomic indicators and COVID-19-associated hospitalization rates—COVID-NET surveillance areas in 14 states, March 1–April 30, 2020
Source: PLoS One. 2021 Sep 24;16(9):e0257622. doi: 10.1371/journal.pone.0257622 (PMC8462704; doi:10.1371/journal.pone.0257622)

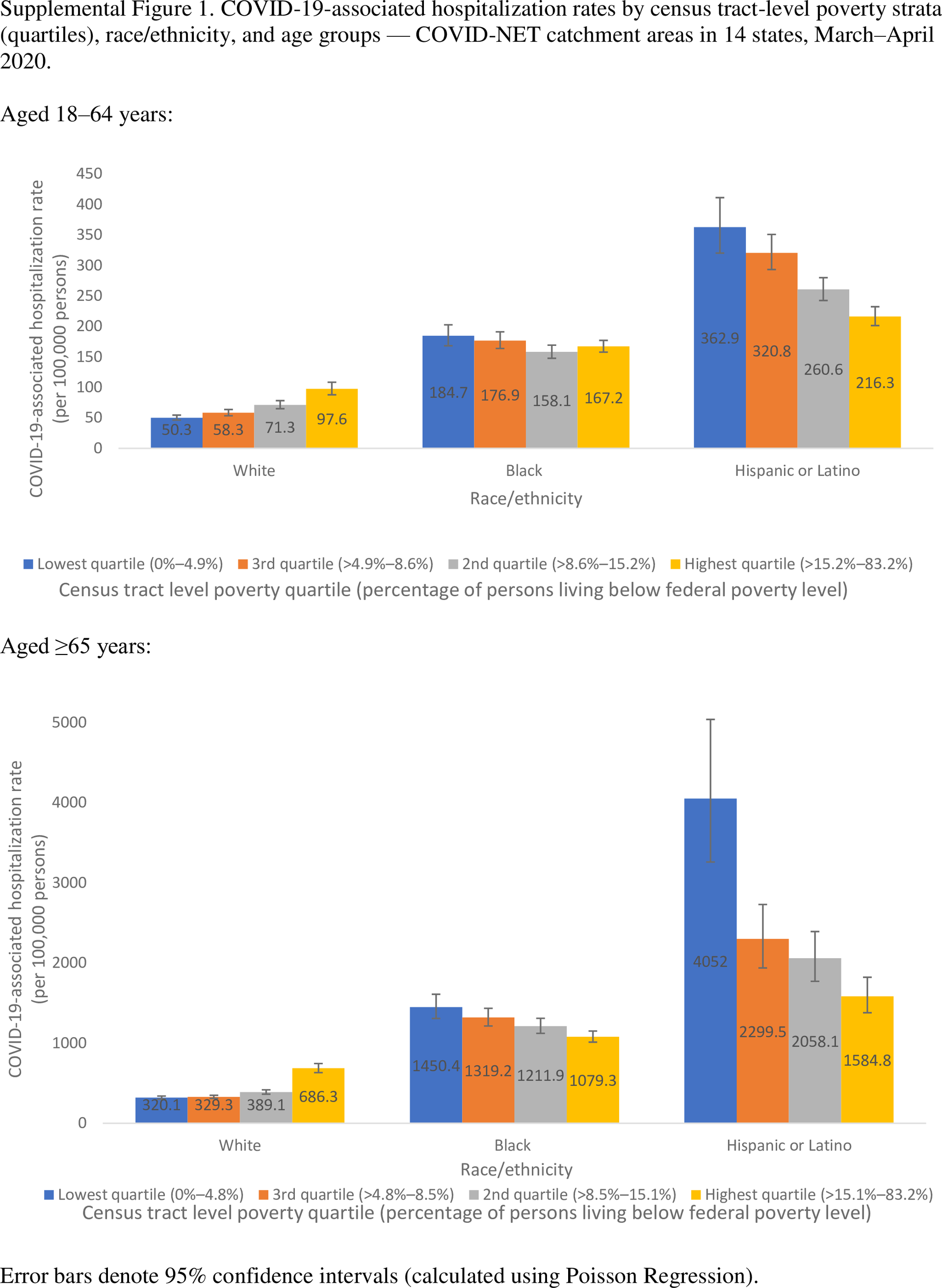

Supplement: S1 Fig — (TIF) [file pone.0257622.s001.tif]

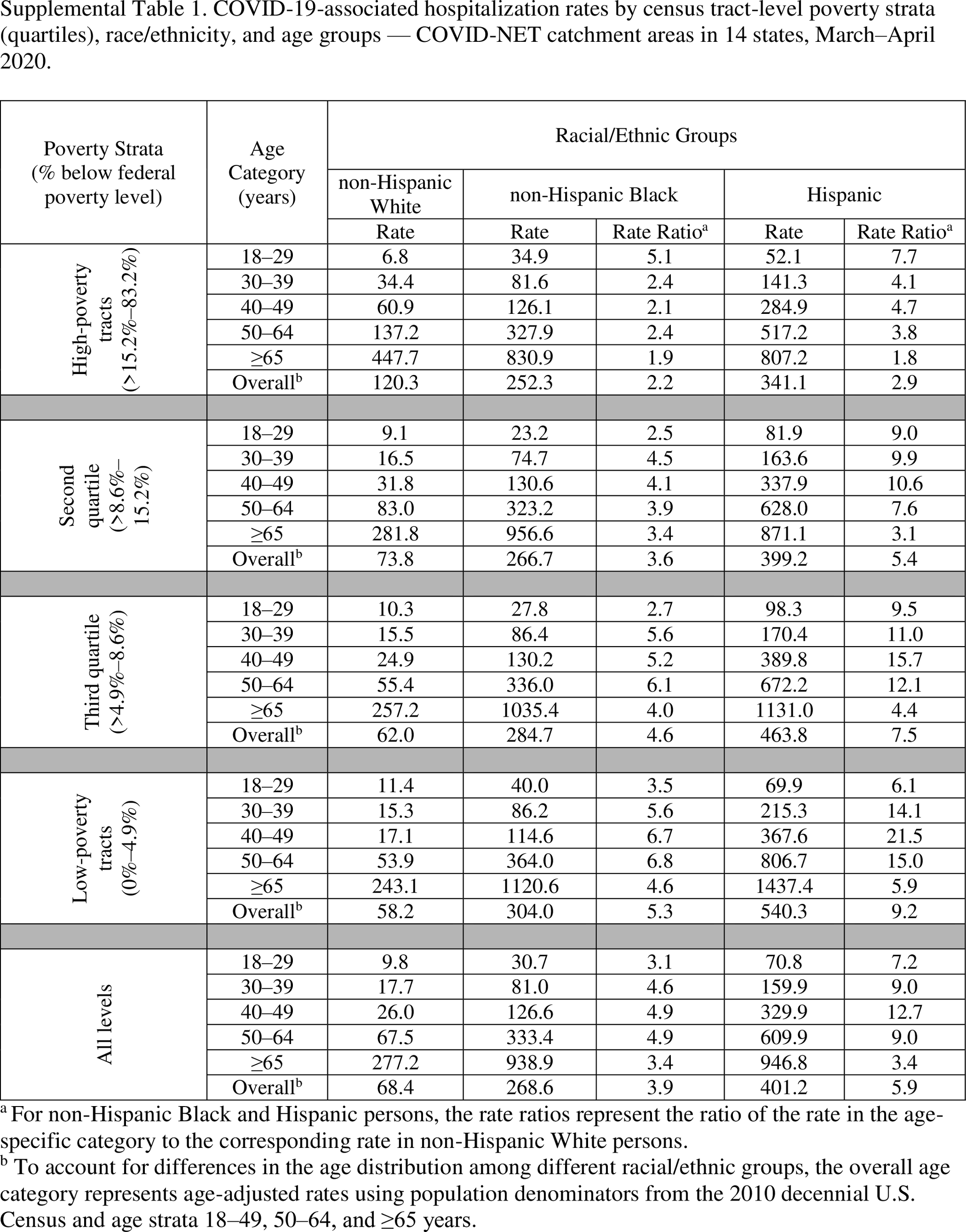

Supplement: S1 Table — (TIF) [file pone.0257622.s002.tif]
